# The association between human blood clot analogue computed tomography imaging, composition, contraction, and mechanical characteristics

**DOI:** 10.1371/journal.pone.0293456

**Published:** 2023-11-13

**Authors:** Janneke M. H. Cruts, Jo-Anne Giezen, Kim van Gaalen, Robert Beurskens, Yanto Ridwan, Marcel L. Dijkshoorn, Heleen M. M. van Beusekom, Nikki Boodt, Aad van der Lugt, Judith J. de Vries, Moniek P. M. de Maat, Frank J. H. Gijsen, Rachel M. E. Cahalane

**Affiliations:** 1 Department of Biomedical Engineering, Erasmus Medical Center, Rotterdam, the Netherlands; 2 Department of Biomechanical Engineering, Delft University of Technology, Delft, the Netherlands; 3 Department of Radiology and Nuclear Medicine, Erasmus Medical Center, Rotterdam, the Netherlands; 4 Department of Molecular Genetics, Erasmus Medical Center, Rotterdam, the Netherlands; 5 Department of Experimental Cardiology, Erasmus Medical Center, Rotterdam, the Netherlands; 6 Department of Neurology, Erasmus Medical Center, Rotterdam, the Netherlands; 7 Department of Public Health, Erasmus Medical Center, Rotterdam, the Netherlands; 8 Department of Hematology, Erasmus Medical Center, Rotterdam, the Netherlands; Foshan Sanshui District People’s Hospital, CHINA

## Abstract

**Background:**

Clot composition, contraction, and mechanical properties are likely determinants of endovascular thrombectomy success. A pre-interventional estimation of these properties is hypothesized to aid in selecting the most suitable treatment for different types of thrombi. Here we determined the association between the aforementioned properties and computed tomography (CT) characteristics using human blood clot analogues.

**Methods:**

Clot analogues were prepared from the blood of 4 healthy human donors with 5 red blood cell (RBC) volume suspensions: 0%, 20%, 40%, 60% and 80% RBCs. Contraction was measured as the weight of the contracted clots as a percentage of the original suspension. The clots were imaged using CT with and without contrast to quantify clot density and density increase. Unconfined compression was performed to determine the high strain compressive stiffness. The RBC content was analysed using H&E staining.

**Results:**

The 5 RBC suspensions formed only two groups of clots, fibrin-rich (0% RBCs) and RBC-rich (>90% RBCs), as determined by histology. The density of the fibrin-rich clots was significantly lower (31-38HU) compared to the RBC-rich clots (72-89HU), and the density increase of the fibrin-rich clots was significantly higher (82-127HU) compared to the RBC-rich clots (3-17HU). The compressive stiffness of the fibrin-rich clots was higher (178–1624 kPa) than the stiffness of the RBC-rich clots (6–526 kPa). Additionally, the degree of clot contraction was higher for the fibrin-rich clots (89–96%) compared to the RBC-rich clots (11–77%).

**Conclusions:**

CT imaging clearly reflects clot RBC content and seems to be related to the clot contraction and stiffness. CT imaging might be a useful tool in predicting the thrombus characteristics. However, future studies should confirm these findings by analysing clots with intermediate RBC and platelet content.

## 1. Introduction

Endovascular thrombectomy (EVT) is proven to be an effective and safe treatment for patients with acute ischemic stroke (AIS) due to a large vessel occlusion and is currently the standard of care [1]. However, further improvement is needed, as the achievement of successful reperfusion is associated with outcome after EVT, yet this successful reperfusion is not achieved in 34–42% of the patients [2–4]. Studies on thrombi retrieved with EVT found that the main components of AIS thrombi are red blood cells (RBCs), fibrin, platelets, and white blood cells (WBCs) [5], and that the thrombus composition is highly variable and an important factor influencing successful reperfusion [6, 7].

Studies with blood clot analogues and human stroke thrombi have shown that the thrombus composition affects the thrombus’ mechanical properties; fibrin-rich thrombi are on average stiffer than RBC-rich thrombi [6, 8–11]. The mechanical properties influence the interaction with the thrombectomy device and therefore possibly the effectiveness of EVT [12, 13]. It is already known that RBC-rich thrombi are associated with higher reperfusion rates compared to fibrin-rich thrombi [6, 14–16]. In addition, the fibrin network is tightened by platelet-driven thrombus contraction, which also affects the thrombus properties such as mechanical stiffness [12, 13] and permeability [17], and therefore may also have an influence on the EVT outcome. A pre-interventional estimation of the thrombus composition and contraction may predict the mechanical properties and is hypothesized to aid in selecting the most suitable thrombectomy device and deployment strategy to ensure higher success rates in each individual patient [18, 19].

Computed Tomography (CT) is the most commonly used imaging modality for diagnosis of AIS and could therefore potentially be useful in predicting the thrombus characteristics [20]. RBC-rich thrombi have already been shown to be associated with a higher density assessed on non-contrast computed tomography (NCCT) and are associated with higher success rates after thrombectomy and reduced intervention time compared to fibrin-rich clots with a lower NCCT density [20–23]. However, studies investigating the relationship between thrombus composition and the perviousness (a proxy for the thrombus permeability) assessed on NCCT and contrast-enhanced computed tomography (CECT) imaging showed conflicting results for which no clear explanation has been found yet [20].

The relationship between CT imaging characteristics and composition has been examined previously, but not for human blood clot analogues. Additionally, the relationship between the imaging characteristics and both mechanics and contraction is unknown [20]. The current study aims to assess the use of CT characteristics as a pre-interventional estimation of the clot type by determining the association between the four aforementioned thrombus characteristics. Considering that the imaging characteristics of clots can be studied in a more controlled manner on the bench compared with the in vivo setting, here we utilise human blood clot analogues.

## 2. Materials and methods

### 2.1 Blood clot analogue preparation

Blood was drawn by venepuncture from 4 healthy human volunteers into BD Vacutainer® sodium citrate tubes. Approval for the use of human material was granted by the Dutch Medical Ethical Testing Committee (METC, NL80622.078.22). Written consent was given by the volunteers. The blood was centrifuged, first at 120 *g* for 20 mins to separate the platelet-rich plasma (PRP), and thereafter at 2000 *g* for 10 mins to separate the RBCs from the platelet-poor plasma (PPP). To prepare the blood clot analogues, the PRP and RBCs were reconstructed in 5 different volumetric RBC percentages (i.e. hematocrits): 0%, 20%, 40%, 60% and 80% (from here on referred to as the five RBC suspensions) (S1A Fig). Coagulation was induced by adding CaCl_2_ (C5670, Sigma-Aldrich) and thrombin (T7009, Sigma-Aldrich) to final concentrations of 17mM and 1U/mL, respectively. The suspension was transferred into syringes and placed vertically in a water bath at 37°C to fully contract overnight. For the 0% and 20% RBC clot types, 3 and 2 clots were made, respectively, to obtain sufficient test material to account for the high degree of clot contraction observed in preliminary tests (S1 Table). All clots were fully contracted and the order and time of each subsequent step in the workflow was kept constant for all donors (S2 Table).

### 2.2 Hemostasis

RBC, WBC, and platelet levels were measured from a whole blood sample using a Coulter Counter (COULTER® AC•T diff™ Analyzer, Beckman Coulter, CA). From the PPP, the fibrinogen level was quantified using a Clauss Assay after undergoing one freeze-thaw cycle. To assess the degree of clot contraction, the weight of the expelled serum was expressed as a percentage of the weight of the total suspension (S1B Fig) [13].

### 2.3 Computed tomography imaging

All clot analogues were imaged using two modalities: micro-CT and clinical CT. The higher resolution of the micro-CT images was used to examine the homogeneity of the clots. The clinical CT scanner was used to mimic a clinical setting to study the clot density and the perviousness (the increase in density after contrast administration).

For the micro-CT, the blood clot analogues were placed in 2ml Eppendorf tubes containing paraffin oil and scanning was performed using the Quantum GX2 imaging system (Perkin-Elmer, Groningen, Netherlands). The paraffin oil was chosen to distinguish the clots from the surrounding fluid due to the large difference in density between clots and oil. Scanning was performed using a 36 mm field of view (FOV) for 2 minutes (90 kV/88 uA), voxel size of 72 μm, slice thickness of 72 μm, and X-ray filter Cu 0.06 mm + Al 0.5 mm. The density values in the thrombi were measured using a region growing algorithm in MATLAB and a histogram was plotted to examine the density distribution within the clot.

After micro-CT imaging, the clots were rinsed three times using Gibco™ Dulbecco’s Modified Eagle Medium (DMEM, high glucose, HEPES, no phenol red, ThermoFisher Scientific, USA). The clots were then placed in new Eppendorf tubes containing DMEM to closer mimic surrounding blood in terms of CT density (S1C Fig). Scanning was performed on a 384 (2x192) slices, dual source, CT scanner (Somatom force; Siemens, Erasmus MC in Rotterdam, Netherlands). The clots were scanned with 100 kVp, a rotation time of 1.0 s, and 0.55 pitch. Image reconstructions were made with a FOV of 60 mm, voxel size of 0.12 mm, slice thickness of 0.5 mm, and Hv40 convolution kernel. First, an NCCT was performed to study the density. Subsequently, an iodinated contrast agent (iodixanol 270 mg I/ml, Visipaque^TM^, GE Healthcare, USA) was added to the DMEM (1:20) and the tubes were inverted to mix the contents. Next, CECT scans were made 5, 10, 15, and 20 minutes after administering the contrast agent to study the density increase. A single observer analysed the CT images (JG). For each clot, three non-overlapping regions of interest (ROI) with 1 mm diameter were defined [24]. Within the three ROIs, the mean density of the clot was measured with ImageJ (1.53k, National Institutes of Health), of which the overall mean per clot for the three ROIs was computed. As a measure of the density increase, the increase in density at each timepoint was quantified by subtracting the average density of the clot on the NCCT scan from the average density on the relevant CECT scan.

### 2.4 Mechanical characterisation

After imaging, the clots were cut into 2 mm sections (S1D Fig). The front and back end regions of the clots were discarded. From each of the four donors, 25 sections were prepared: five of each clot type (S1 Table). Four of the five sections from each clot type were used for mechanical testing and one section was set aside for histological analysis (see Section 2.5). To measure the cross-sectional area, the 2 mm sections were photographed next to a ruler, so that the cross-sectional area could be measured using ImageJ. After photographing each section, it was placed on the stage of the compression tester in a 37°C water bath filled with DMEM. Compression tests were performed using a custom made compression tester, as previously described (S1E Fig) [8]. The sections were compressed to 80% strain, with a compression and retraction speed of 0.2 mm/s (10% strain/s) [8, 25]. The measured force was converted to stress using the cross-sectional surface area of the samples. As a measure for the stiffness, the high strain secant moduli were calculated from the slopes of fitted straight lines to the final 2% linear portion of the nominal stress-strain curves [13]. For each donor, the average high strain stiffness of the four sections per clot type was computed.

### 2.5 Histology

It is known that the volumetric percentage of RBCs in ovine blood suspensions does not result in a clot with an equivalent proportion of RBCs [26]. Therefore, the composition of the clots in the current study was confirmed using histology (S1F Fig). The sections were placed in buffered 4% formaldehyde for 12 hours at 4°C, embedded in paraffin wax and sectioned using 5 μm slice thickness. Hemotoxylin and Eosin (H&E) stain was used to identify the blood clot analogue components. The samples were then scanned at 40x magnification and 0.23 μm/pixel resolution (2.0 HT Nanozoomer, Hamamatsu, Japan). The percentage areas of RBCs were quantified using Orbit Image Analysis software [27], of which the average of the two slices was computed.

### 2.6 Statistical analysis

Shapiro-Wilk tests were performed and histogram plots were visualized for each of the four clot characteristics to examine if the data within each clot type was normally distributed. If the data for the majority of the clot types was normally distributed, one-way ANOVA tests were performed to evaluate differences between clot types, followed by post-hoc Tukey’s HSD tests. If the data for the majority of the clot types was not normally distributed, Kruskal-Wallis H tests were performed, followed by pairwise comparisons. Within one clot type, t-tests were performed to assess differences in density between NCCT and CECT scans after 20 minutes. A P-value lower than 0.05 was considered statistically significant. All statistical tests were performed using IBM SPSS Statistics 28.

## 3. Results

### 3.1 Donor and sample details

A total of 4 healthy donors were included in the current study (3 males, 1 female, age range 23–54 years old). The whole blood cell counts and fibrinogen levels can be found in S3 Table. In total, 33 analogue clots were created (S1 Table), for which the imaging characteristics and degree of contraction were quantified. Subsequently, the clots were cut into a total of 78 sections for mechanical testing and 20 sections for histological analysis.

### 3.2 Composition

**Fig 1:**
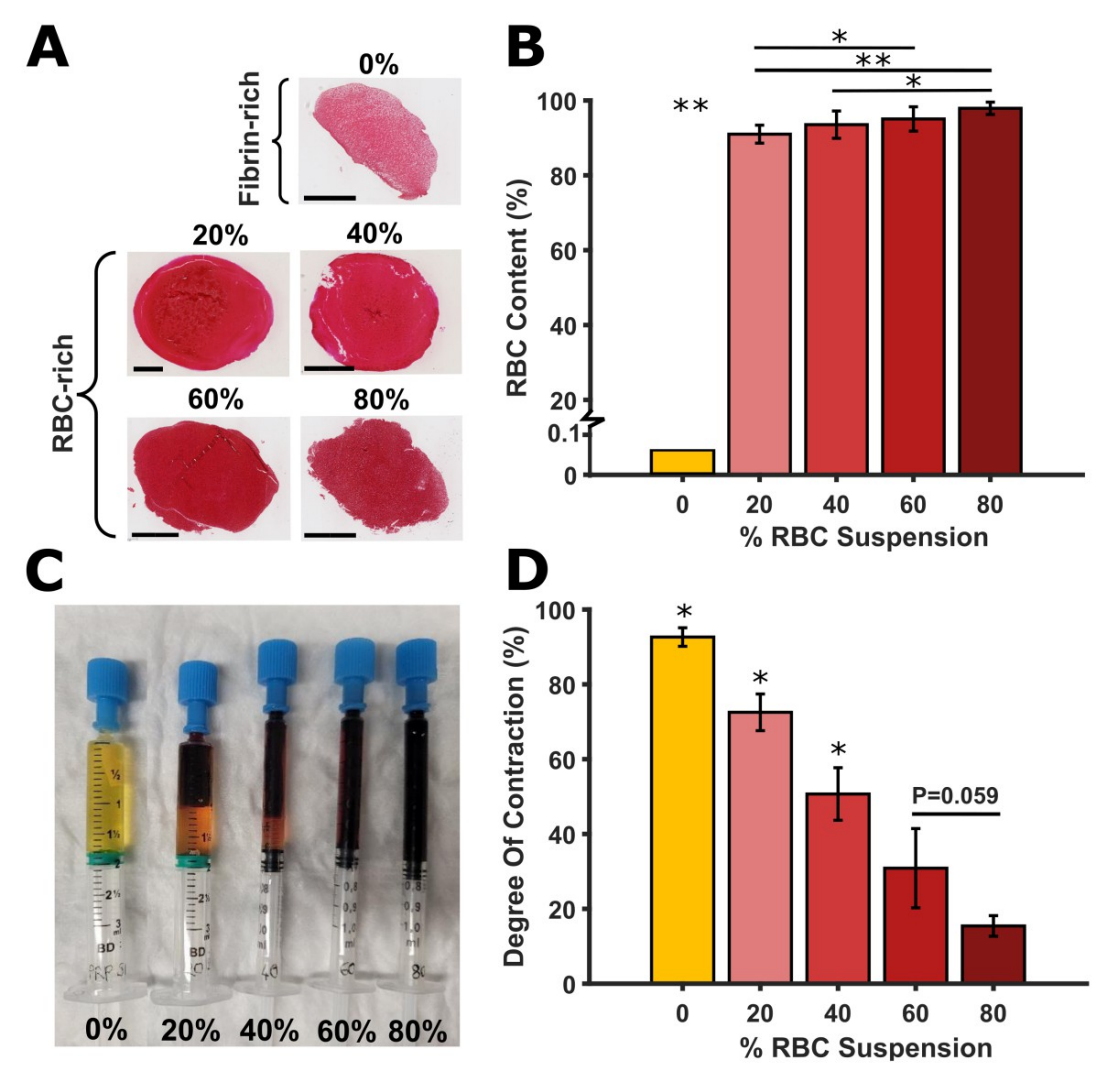
Blood clot analogue red blood cell (RBC) content and contraction. (A) Representative hematoxylin and eosin (H&E) stain slices of the five RBC suspensions. The 0% RBC suspension produced fibrin-rich clots (0% RBCs) and the 20%, 40%, 60%, and 80% RBC suspensions produced RBC-rich clots (>90% RBCs). All scale bars represent 1mm. (B) RBC content in the blood clot analogues vs RBC suspension (%). (C) Contracted clots of the five RBC suspensions with expelled serum. (D) Degree of contraction vs RBC suspension (%). Statistical significance determined using one-way ANOVA: *P < 0.05, **P < 0.001 (* without a line means significant compared to all others).

Composition analysis showed that the initial RBC suspensions of 0%, 20%, 40%, 60% and 80% RBCs produced clots with non-equivalent compositions of 0.06 ± 0.09%, 91 ± 2%, 94 ± 4%, 95 ± 3%, and 98 ± 2% RBCs, respectively (Fig 1A and 1B). This indicates that only fibrin-rich clots (0% RBCs) and RBC-rich clots (>90% RBCs) were produced. Furthermore, it was observed that the composition of all clots were generally homogeneously distributed, but often contained more fibrin on the edge as expected for contracted clot analogues (S2 Fig) [28]. The data per donor can be found in S3 Fig. The fibrin/platelet and WBC content can be found in S4 Table.

### 3.3 Contraction

The degree of clot contraction was observed to decrease with increasing RBC volume in the original suspension (Fig 1C and 1D). A significant difference in degree of contraction was observed between all the clot types (all P<0.05), except between the 60% and 80% RBC suspension clots (P = 0.059). The data per donor can be found in S4 Fig.

### 3.4 Mechanics

**Fig 2:**
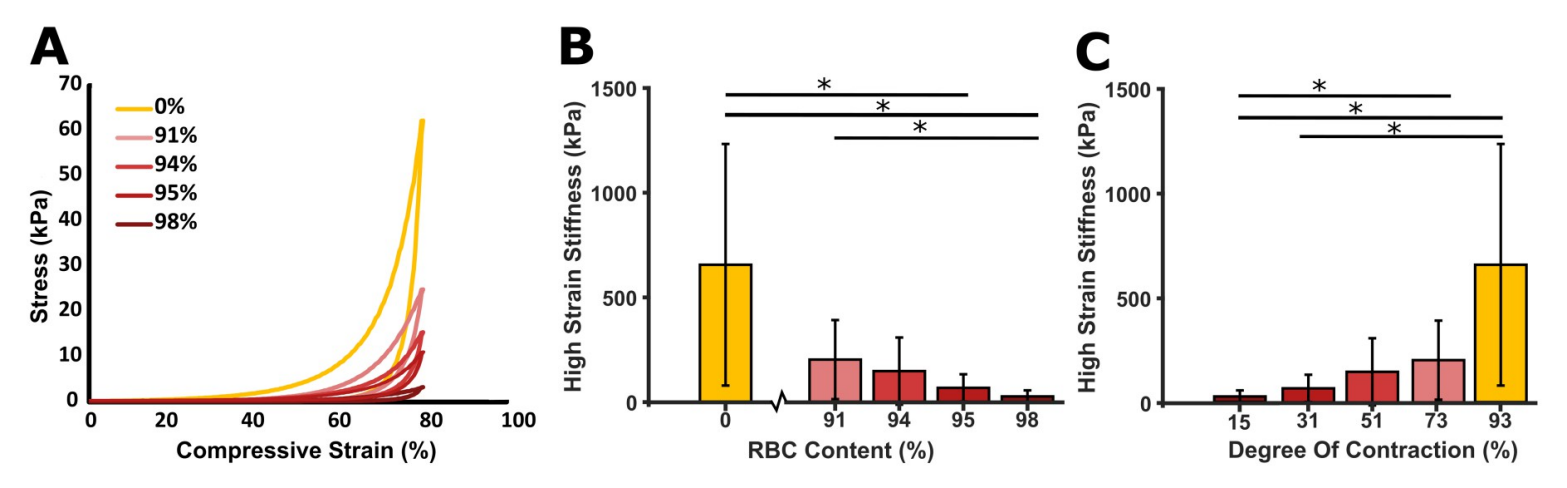
Blood clot analogue mechanical properties. (A) Example stress-strain loading and unloading curve for the five RBC-content clots. (B) Mean high strain stiffness vs RBC content (%). (C) Mean high strain stiffness vs degree of contraction (%). Statistical significance determined using Kruskal-Wallis H: *P < 0.05, **P < 0.001 (* without a line means significant compared to all others).

In total, 78 sections were mechanically tested (S1 Table). It was observed that all clots exhibit non-linear stress-strain behaviour (Fig 2A). A statistically significant difference in high strain stiffness was found between the RBC-content clots 0%-95%, 0%-98%, and 91%-98% (P<0.05), but not between the other RBC-content clots (P-values ranging from 0.094 to 0.55) (Fig 2B). The data per donor can be found in S5 Fig. For each donor the same behavior was observed: a decrease in stiffness with increasing RBC content. The stiffness also increased with increasing degree of contraction (Fig 2C).

### 3.5 Computed tomography characteristics

**Fig 3:**
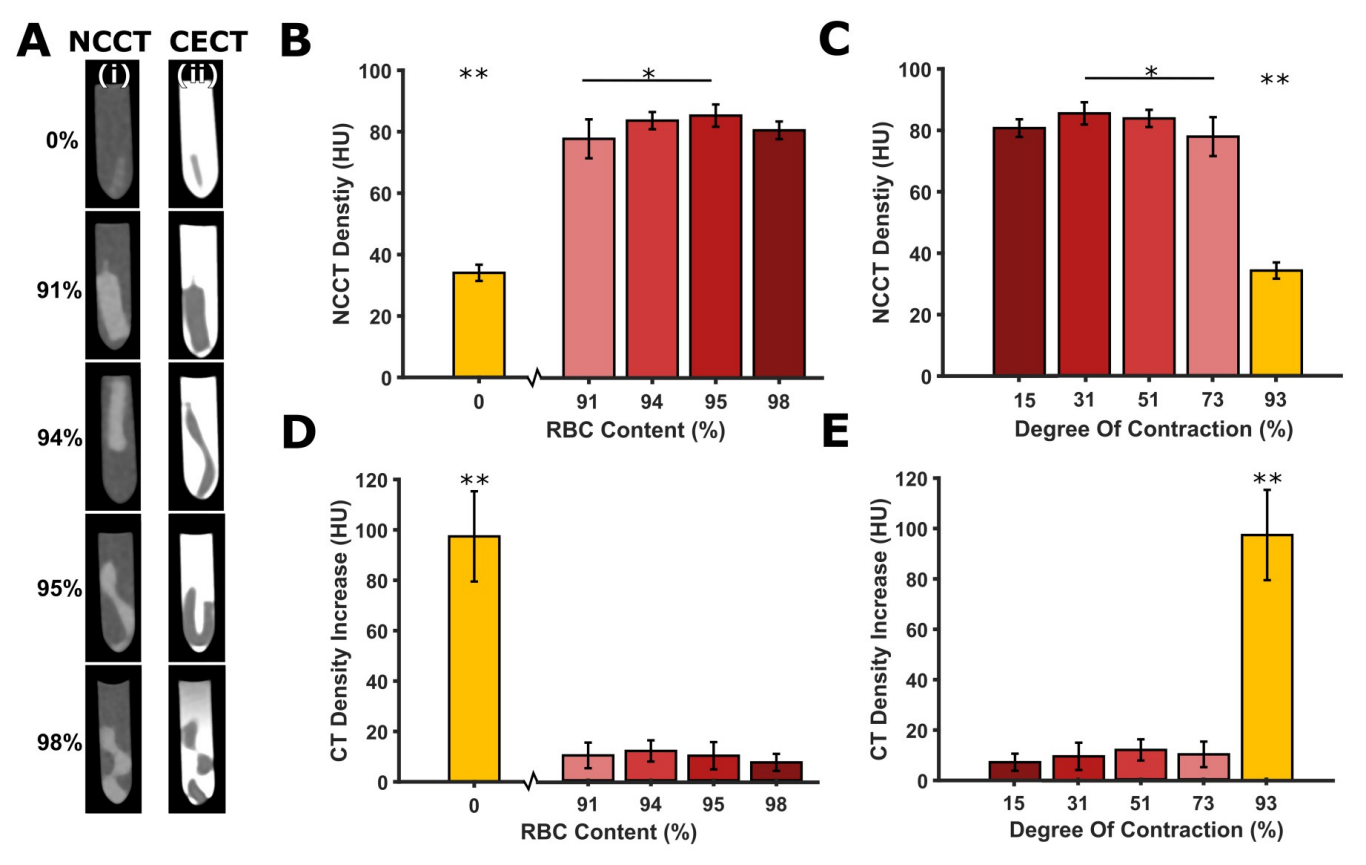
Blood clot analogue imaging characteristics. (A) Clinical computed tomography (CT) images of the five analogue RBC-content clots in the Eppendorf tubes, with the (i) non-contrast CT (NCCT) images and the (ii) contrast-enhanced CT (CECT) images 20 minutes after contrast administration. (B) Mean NCCT densities vs RBC content (%). (C) Mean NCCT densities vs degree of contraction (%). (D) Mean CT density increase 20 minutes after contrast administration vs RBC content (%). (E) Mean CT density increase 20 minutes after contrast administration vs degree of contraction (%). Statistical significance determined using one-way ANOVA: *P < 0.05, **P < 0.001 (* without a line means significant compared to all others).

A representative micro-CT image is presented in S6 Fig. A normally distributed histogram of micro-CT density values was observed for each of the 5 clot types for each donor (S7 Fig), confirming the homogeneity of the clot analogues in the current study. In addition, the clot homogeneity is studied by analysing the density variations along the longitudinal axis, which demonstrates that the density remains relatively consistent throughout its length (S8 Fig). Notably, lower densities were observed at the front and back ends of the clot; however, these clot regions were discarded during clot sectioning (Section 2.4, S1D Fig) and were excluded from the CT analysis by placing the ROIs away from the clot edge. The NCCT density of the fibrin-rich clots was significantly lower compared to the density of the RBC-rich clots (all P<0.05) (Fig 3B). This is in line with the findings of the clot composition analysis. The NCCT densities between the 91%, 94%, 95% and 98% RBC-content clots did not significantly differ from each other (all P>0.05), except for the 91% and 95% RBC-content clots (P = 0.023). The fibrin-rich clots with the highest degree of contraction (93%) had a significantly lower NCCT density than the RBC-rich clots with a lower degree of contraction varying between 15% and 73% (P<0.05) (Fig 3C). Results of the CECT density after 5, 10, 15, and 20 minutes of contrast exposure are shown in S9 Fig. An increase in density was seen for all timepoints for the fibrin-rich clots, but not for all timepoints for the RBC-rich clots. The increase in density after 20 minutes of contrast exposure was significantly higher for the fibrin-rich clots compared to the RBC-rich clots (all P<0.05) (Fig 3D). The same trend can be observed for the degree of contraction: the fibrin-rich clots with a high degree of contraction (mean value of 93%) showed a high density increase, whereas the RBC-rich clots with a lower degree of contraction (mean values between 15 and 73%) showed a lower density increase. The data per donor can be found in S10 and S11 Figs.

### 3.6 Relationship between the clot properties and imaging characteristics

**Fig 4:**
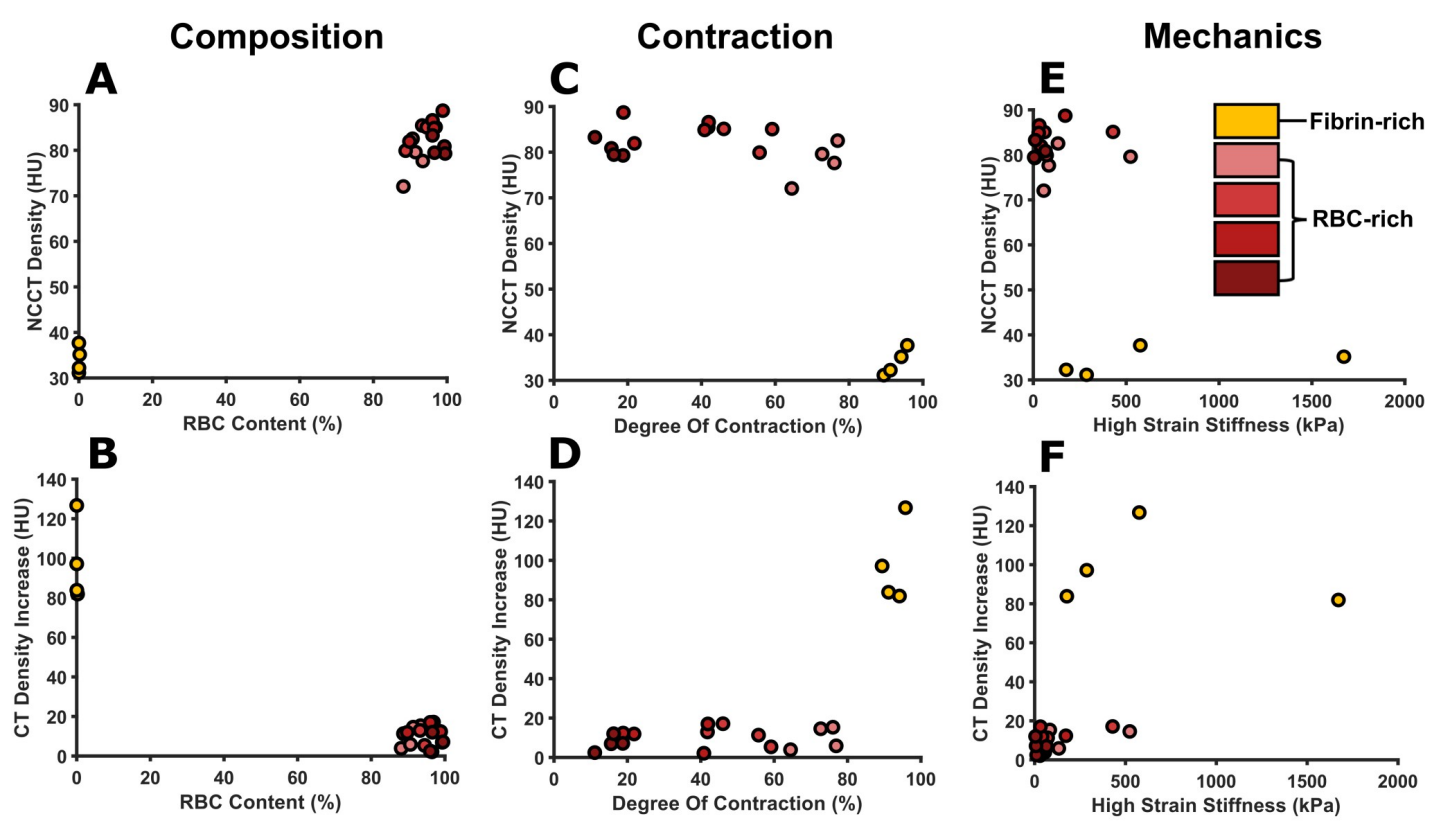
Scatterplots of the clot properties and CT imaging characteristics. Red blood cell (RBC) content versus (A) density and (B) density increase. Degree of contraction versus (C) density and (D) density increase. High strain compressive stiffness versus (E) density and (F) density increase. Yellow and shades of red indicate the fibrin-rich (0% RBCs) and the RBC-rich (>90% RBCs) clots, respectively.

The resulting compositions of the contracted clots are clearly reflected in the imaging characteristics (Fig 4A and 4B): the RBC-rich clots have a significantly higher NCCT density (72-89HU) compared to the fibrin-rich clots (31-38HU). Also, the RBC-rich clots have a lower density increase (3-17HU) compared to the fibrin-rich clots (82-127HU). However, the small differences between the RBC-rich clot compositions were not reflected in significant differences between the density or density increase. Due to the lack of clots with an intermediate RBC content no correlation tests could be performed. The degree of contraction shows a different relationship with the imaging characteristics (Fig 4C and 4D). The RBC-rich clots with a varying degree of contraction (11–76%) show a relatively constant NCCT density (72-89HU); however, a slight decrease in density with increasing contraction can be observed. The fibrin-rich samples are contracted most (89–96%) and show a significantly lower NCCT density compared to the RBC-rich clots (31-38HU). The opposite observation was made for the density increase; the RBC-rich clots with a varying degree of contraction (11–76%) all show a constant low density increase (3-17HU), whereas the fibrin-rich clots with highest degree of contraction (89–96%) show a high density increase (82-127HU). Regarding the mechanical properties, the RBC-rich clots tend to cluster around low stiffness (6–526 kPa) and high NCCT values, while the fibrin-rich clots cover a large range of stiffness values (178–1624 kPa) at much lower NCCT values (Fig 4E and 4F). This also holds for the density increase, for which the RBC-rich clots cluster around low stiffness values (6–526 kPa) and a low density increase, whereas the fibrin-rich clots are spread over a wider range of stiffness values (178–1624 kPa) at a much higher density increase. Of note, the standard deviation for the compressive high strain stiffness between donors was much higher compared to the standard deviation of the clot RBC content, contraction and imaging characteristics. However, for each donor the same trend was observed: the high strain stiffness decreased with increasing RBC content, and the stiffness of the fibrin-rich clots was at least twice as high as that of the RBC-rich clots (S5 Fig).

## 4. Discussion

The aim of this study was to investigate the association between human blood clot analogue computed tomography imaging, composition, contraction, and mechanical characteristics. The clot analogues preparation protocol used in the current study produced fibrin-rich (0% RBCs) and RBC-rich clots (>90% RBCs). On CT imaging the fibrin-rich clots showed a low NCCT density and a high density increase, whereas all RBC-rich clots resulted in a high NCCT density and low density increase. The RBC-rich clots with a varying degree of contraction showed a constant NCCT density and density increase; however, the fibrin-rich clots with the highest degree of contraction showed a lower NCCT density and higher density increase. The high strain stiffness of the fibrin-rich clots was substantially higher than the stiffness of the RBC-rich clots.

We showed that the percentage RBCs in the original suspensions did not result in clots with an equivalent RBC content. This finding can possibly be explained by the fact that due to contraction most RBCs will be trapped into the fibrin network. The incompressible nature of RBCs limits the extent of contraction. A consequence of this is that higher hematocrits will lead to larger clots. As demonstrated in the current study and others, RBCs will fill the majority of the clot, leading to clots with >90% RBCs for RBC suspensions as low as 20% [29]. Duffy et al. also found non-equivalence between the RBC content of the suspension and the contracted clot. For an ovine 40% RBC suspension, clots contained 68 ± 4% RBCs [26]. However, for a human 40% RBC suspension, clots contained 94 ± 4% RBCs (current study). This discrepancy in RBC content for similar RBC suspensions could be caused by differences between the RBC size in humans versus sheep [30].

The composition of clot analogues significantly alters the density of the clot on NCCT scans. Qualitatively, the presence of a hyperdense middle cerebral artery sign (HMCAS) in patients with AIS is reflective of RBC-rich thrombi. These RBC-rich clots have a higher density than the fibrin-rich clots [20]. It is likely related to the fact that in CT imaging the density of the tissue is proportional to the attenuation of the X-rays passing through, and RBCs are large incompressible cells with a relatively high density compared to the other blood components [31]. In addition, CT density is linearly correlated with the concentration of haemoglobin [32]. These findings are clinically relevant, since the NCCT density and RBC content have been shown to be associated with the success rates and procedure time of thrombectomy. Patients presenting with HMCAS (RBC-rich thrombi) have been reported to require a greater number of thrombus passes and experience longer procedure times [33].

Only few prior studies have directly investigated the association between clot perviousness (density increase) based on CT density and the histologic composition of clots, of which the results were conflicting [20]. Berndt et al., Patel et al., and Wei et al. found a negative correlation between the perviousness and RBC content [34–36], whereas Benson et al. found a positive correlation [21]. The reason that Benson et al. found an opposite correlation could be because of their statistical analysis, which did not directly study the correlation between composition and perviousness. In this study, we observed a negative correlation with the RBC content, since the density increase was lower for the RBC-rich clots and higher for the fibrin-rich clots, which is in accordance with most of the other studies. This suggests that RBCs impede the penetration of the contrast agent and that therefore the contrast agent can more easily penetrate the fibrin-rich clots compared to the RBC-rich clots. Furthermore, the higher density increase of the fibrin-rich clots could be caused by the intrinsic affinity of fibrin proteins to iodine which is present in the contrast agent [37].

The density increase is a proxy for the clot’s permeability [21], which is hypothesised to be influenced by the degree of contraction. Due to contraction the RBCs change to a polyhedrocyte shape, which has been postulated to create impermeability of the clot due to the more densely packed RBCs [28]. However, although the degree of contraction was found to be hematocrit-dependent, the density increase (and NCCT) of the RBC-rich clots did not significantly differ. It seems that for CT imaging the RBC content dominates over the degree of contraction (Fig 4A–4D), and therefore the density increase is more dependent on the composition. This explains why no clear relationship between the density increase and contraction can be observed for the 91–98% RBC-content clots. Conceivably, a threshold of RBC-concentration in the reconstructed blood exists at which the clot will be optimally packed after contraction. Beyond this threshold differences in degree of contraction might not influence the internal structure or permeability of the clot. Furthermore, due to the nature of the clot preparation using different volumes of PRP in the original suspension, each RBC suspension clot had a different concentration of platelets. Further research on this topic is required to elucidate the effect of both platelet and RBC concentrations on the clot contraction, internal structure, permeability and density increase.

It was observed that the stiffness of the clots decreased with increasing RBC content, which implies that the mechanical properties of the clot analogues were found to be hematocrit-dependent. A possible explanation for this is that with a lower hematocrit more fibers can attach to and be tightened by the platelets. RBC-rich clots also contain a looser network of thin fibrin strands, whereas thick fibers forming bundles are present in fibrin-rich clots [38]. Alternatively, the differences in concentrations of platelets (as described above) could be causing the differences in degree of contraction and resistance to deformation (stiffness). The RBC-rich clots were clustered around relatively low stiffness values at a high NCCT density and low density increase, whereas the fibrin-rich clots covered both low and relatively high stiffness values at a low NCCT density and high density increase. An even clearer distinction between the stiffness of the fibrin-rich clots and the RBC-rich clots was observed per donor (S5 Fig), with the fibrin-rich clots being at least twice as stiff as the RBC-rich clots. One could state therefore that in this study relatively high stiffness values were more reflected by a low NCCT density and high density increase. Thus, by identifying the fibrin-rich clots by density increase analyses we could get evidence for the stiffness. When looking at Fig 4E and 4F, it is expected that a correlation between CT imaging characteristics and stiffness will be found in analyses of more clots with varying fibrin. However, it must be noted that previous studies showed that 5% and 20% RBC suspension clots have higher stiffness than 0% RBC suspension clots [13]. The relationship between imaging and mechanics thus remains a topic for future research in which clots with intermediate RBC content should be investigated and confirmed with histology.

This study has limitations that should be acknowledged. First, only fibrin-rich and RBC-rich clots were produced. Future research should focus on preparing clots with an intermediate RBC content to accurately reflect the range of RBCs in thrombi retrieved from EVT [39]. This will ensure the ability to quantify the correlation between the four clot characteristics analysed in this study. Furthermore, although our in vitro setup was designed to mimic the clinical setting as closely as possible, differences with the in vivo situation should be considered. First of all, any tissue surrounding the clot in a patient could alter the CT attenuation characteristics of the clot, which are not taken into account in our in vitro setup. However, DMEM can closely simulate the blood density (12HU vs 15-50HU, for DMEM and blood, respectively). Second, the perviousness (density increase) was studied in a static condition without incorporating the effects of blood flow which are present in the clinical setting. Third, the clots were formed statically which results in a homogeneous structure, whereas in patients the clots are formed under flow which can cause heterogeneity in the composition of in vivo clots [39]. However, to reduce the number of variables in the mechanical and imaging characterisation elements of this study we used homogeneous clot analogues. Fourth, in vivo clots may be formed within a shorter or longer timeframe prior to the onset of acute ischemic stroke. To limit the effects of any age-related clot analogues changes we used a consistent experimental schedule for clot formation (a fixed 24-hour time frame) and all subsequent characterisations. Future research should examine the association between mechanical and CT imaging characteristics of thrombi from thrombectomy procedures, in contrast to human blood clot analogues derived from healthy volunteers. In this way, we could confirm if the findings from the current study can be related to the clinical setting. Also, while our study provides valuable insights, the limited sample size should be acknowledged. Although minimal differences were observed in most clot characteristics between donors, significant variations were noted in the mechanical properties. Future research should therefore consider replicating our study with a larger sample size to validate our findings on a broader scale. Lastly, it should be noted that the clot characteristics investigated in this study are not the only variables known to influence the outcome of the thrombectomy procedures. Other important clinical variables related to the thrombus aside from histopathology include the occlusion location and thrombus length [40].

## 5. Conclusion

In this study the five RBC suspensions resulted in two clot groups: fibrin-rich and RBC-rich, which was reflected by the CT imaging characteristics. The fibrin-rich clots showed a low NCCT density and a high density increase, whereas the RBC-rich clots showed a high NCCT density and low density increase. The fibrin-rich clots showed a higher degree of contraction and compressive stiffness than the RBC-rich clots. Therefore, it can be suggested that CT imaging might be a useful tool in predicting the thrombus characteristics. However, future studies should focus on the production of clots with intermediate RBC content and different concentrations of platelets to validate these findings.

## Supporting information

S1 Table Overview of the total amount of blood clot analogues per donor, the total amount of sections cut from these clots, the amount of sections used for mechanics and composition analysis, and the amount of slices (cut from the “histology section”) used for histology. All clots were used for both computed tomography (CT) imaging and the measurement of the degree of contraction. The resulting clot characteristic values were averaged (by the numbers presented in bold), resulting in only one value per clot type per donor.
(DOCX)

S2 Table Time schedule for the clot analogue preparation, computed tomography (CT) imaging, compression testing and fixation for histology. All steps were taken the same for each donor.
(DOCX)

S3 Table Donor whole blood cell counts and fibrinogen levels. (DOCX)

S4 Table Red blood cell (RBC), fibrin/platelets, and white blood cell (WBC) content in the contracted clots for the 0%, 40%, 20%, 60%, and 80% RBC suspensions. The mean value ± standard deviation are presented.
(DOCX)

S1 Fig Overview of the experimental workflow. (A) Blood clot analogue preparation. Whole blood (WB) is spun in a centrifuge to separate platelet rich plasma (PRP), platelet poor plasma (PPP) and the red blood cells (RBCs). Five RBC suspensions are created using PRP with a RBC concentration of 0%, 20%, 40%, 60%, and 80%. (B) Clot contraction measurements. The empty syringe, syringe with contracted clot and serum, and separate clot or serum are weighed. (C) Computed tomography (CT) imaging. A scan of the clot in an Eppendorf tube is made, after which contrast is administered and more scans are made after specific time points. (D) The clot is cut into 5 sections: 4 for mechanical testing and 1 for composition analysis. The ends of the clot are discarded. (E) Mechanical testing. The clot is compressed up to 80% strain in a custom-made compression tester. (F) Composition analysis. The clot section is embedded and sliced in two slices. Finally, the slices are stained using H&E and the RBC content is quantified. Sections of this figure were created with BioRender.com.
(TIF)

S2 Fig Representative H&E staining of a 20% red blood cell (RBC) volume clot. (A) An overview. (B) Magnified image which demonstrates that the clot consists primarily of RBCs; however, more fibrin tends to be present on the edge of clots compared to the internal portion.
(TIF)

S3 Fig Red blood cell (RBC) content in the blood clot analogues vs RBC suspension per donor. (TIF)

S4 Fig Clot analogue degree of contraction vs red blood cell (RBC) suspension per donor. (TIF)

S5 Fig Clot analogue mean high strain stiffness vs red blood cell (RBC) content per donor. (TIF)

S6 Fig Representative micro computed tomography (micro-CT) scan of a clot analogue surrounded by paraffin oil in an Eppendorf tube. (TIF)

S7 Fig Histograms of the micro computed tomography (micro-CT) density values for the five clot types. (A) donor 1, (B) donor 2, (C) donor 3, (D) donor 4. The normalized density histograms were acquired using an automatic binning algorithm based on Sturge’s rule: *k* = ⌈1 + log_2_ *n*⌉, where *k* is the number of bins and *n* is the number of observations.
(TIF)

S8 Fig Micro computed tomography (micro-CT) density along the clot’s longitudinal axis. (A) Example micro-CT image of a clot with the indicated line over which the CT density is measured. (B) The distance of a clot (from top to bottom) vs the CT density for the five clot types of donor 2.
(TIF)

S9 Fig Clot analogue mean computed tomography (CT) density on the non-contrast CT (NCCT) scans and on the contrast-enhanced CT (CECT) scans, 5, 10, 15 and 20 minutes after the administration of the contrast agent. The error bars represent the standard deviation. The density significantly increased after 20 minutes of contrast exposure compared to the NCCT scan for the clots with RBC-content of 0%, 91% and 95% (P-values ranging from P = 0.003 to P = 0.043), except for the clots with RBC-content of 94% and 98% (P-value of 0.068 and 0.098, respectively).
(TIF)

S10 Fig Clot analogue mean non-contrast computed tomography (NCCT) densities vs red blood cell (RBC) content per donor. (TIF)

S11 Fig Clot analogue mean computed tomography (CT) density increase 20 minutes after contrast administration vs red blood cell (RBC) content per donor. (TIF)

S1 Raw data (XLSX)
