# Supplementary material for: The association between human blood clot analogue computed tomography imaging, composition, contraction, and mechanical characteristics
Source: PLoS One. 2023 Nov 13;18(11):e0293456. doi: 10.1371/journal.pone.0293456 (PMC10642823; doi:10.1371/journal.pone.0293456)
Supplement: S1 Table — All clots were used for both computed tomography (CT) imaging and the measurement of the degree of contraction. The resulting clot characteristic values were averaged (by the numbers presented in bold), resulting in only one value per clot type per donor. (DOCX) [file pone.0293456.s001.docx]

| **Donor** | **RBC suspension** | **Total amount of clots**  **(for CT**  **and**  **contraction)** | **Total amount of sections** | **Sections for**  **compression** | **Sections for histology** | **Slices for histology** |
| --- | --- | --- | --- | --- | --- | --- |
| **1** | 0% | **3** | 5 | **4** | 1 | **2** |
|  | 20% | **2** | 5 | **4** | 1 | **2** |
|  | 40% | **1** | 5 | **4** | 1 | **2** |
|  | 60% | **1** | 5 | **4** | 1 | **2** |
|  | 80% | **1** | 4 | **3** | 1 | **2** |
| **2** | 0% | **3** | 5 | **4** | 1 | **2** |
|  | 20% | **2** | 5 | **4** | 1 | **2** |
|  | 40% | **1** | 5 | **4** | 1 | **2** |
|  | 60% | **1** | 5 | **4** | 1 | **2** |
|  | 80% | **2** | 5 | **4** | 1 | **2** |
| **3** | 0% | **3** | 4 | **3** | 1 | **2** |
|  | 20% | **2** | 5 | **4** | 1 | **2** |
|  | 40% | **1** | 5 | **4** | 1 | **2** |
|  | 60% | **1** | 5 | **4** | 1 | **2** |
|  | 80% | **1** | 5 | **4** | 1 | **2** |
| **4** | 0% | **3** | 5 | **4** | 1 | **2** |
|  | 20% | **2** | 5 | **4** | 1 | **2** |
|  | 40% | **1** | 5 | **4** | 1 | **2** |
|  | 60% | **1** | 5 | **4** | 1 | **2** |
|  | 80% | **1** | 5 | **4** | 1 | **2** |
