# Supplementary material for: The association between human blood clot analogue computed tomography imaging, composition, contraction, and mechanical characteristics
Source: PLoS One. 2023 Nov 13;18(11):e0293456. doi: 10.1371/journal.pone.0293456 (PMC10642823; doi:10.1371/journal.pone.0293456)
Supplement: S2 Table — All steps were taken the same for each donor. (DOCX) [file pone.0293456.s002.docx]

| **Day** | **Time (hour)** | **Step** |
| --- | --- | --- |
| 1 | 09:00-12:00 | Sample preparation |
| 1-2 | 12:00 (Day 1) – 13:00 (Day 2) | Sample incubation at 37°C |
| 2 | 13:00-14:00 | Sample processing for micro CT |
| 2 | 14:00-16:00 | Micro CT imaging |
| 2 | 16:00-17:00 | Sample processing for clinical CT |
| 2 | 17:00-18:00 | Clinical CT imaging |
| 2 | 19:00-23:00 | Compression testing |
| 2 | 19:00-23:00 | Sample fixation for histological analysis |
