# Supplementary material for: The association between human blood clot analogue computed tomography imaging, composition, contraction, and mechanical characteristics
Source: PLoS One. 2023 Nov 13;18(11):e0293456. doi: 10.1371/journal.pone.0293456 (PMC10642823; doi:10.1371/journal.pone.0293456)
Supplement: S3 Table — (DOCX) [file pone.0293456.s003.docx]

| **Donor ID** | **Red**  **Blood Cells**  **(x 10^3^ cells/μL)** | **White Blood Cells**  **(x 10^3^ cells/μL)** | **Platelets**  **(x 10^3^ cells/μL)** | **Fibrinogen**  **(g/L)** |
| --- | --- | --- | --- | --- |
| 1 | 4.71 | 4.7 | 194 | 2.0 |
| 2 | 4.91 | 4.1 | 236 | 2.3 |
| 3 | 4.37 | 4.0 | 244 | 2.2 |
| 4 | 4.56 | 7.3 | 216 | 3.6 |
