# Supplementary material for: The association between human blood clot analogue computed tomography imaging, composition, contraction, and mechanical characteristics
Source: PLoS One. 2023 Nov 13;18(11):e0293456. doi: 10.1371/journal.pone.0293456 (PMC10642823; doi:10.1371/journal.pone.0293456)
Supplement: S4 Table — The mean value ± standard deviation are presented. (DOCX) [file pone.0293456.s004.docx]

|  | **0%** | **20%** | **40%** | **60%** | **80%** |
| --- | --- | --- | --- | --- | --- |
| **RBCs** | 0.063 ± 0.095 | 91 ± 2.4 | 94 ± 3.6 | 95 ± 3.3 | 98 ± 1.6 |
| **Fibrin/Platelets** | 99 ± 0.78 | 9.7 ± 2.4 | 4.4 ± 3.6 | 3.0 ± 3.2 | 0.43 ± 2.4 |
| **WBCs** | 1.2 ± 0.69 | 0.28 ± 2.4 | 0.19 ± 0.27 | 0.04 ± 1.2 | 0.068 ± 0.040 |
