# Supplementary figures and images for: The association between human blood clot analogue computed tomography imaging, composition, contraction, and mechanical characteristics
Source: PLoS One. 2023 Nov 13;18(11):e0293456. doi: 10.1371/journal.pone.0293456 (PMC10642823; doi:10.1371/journal.pone.0293456)

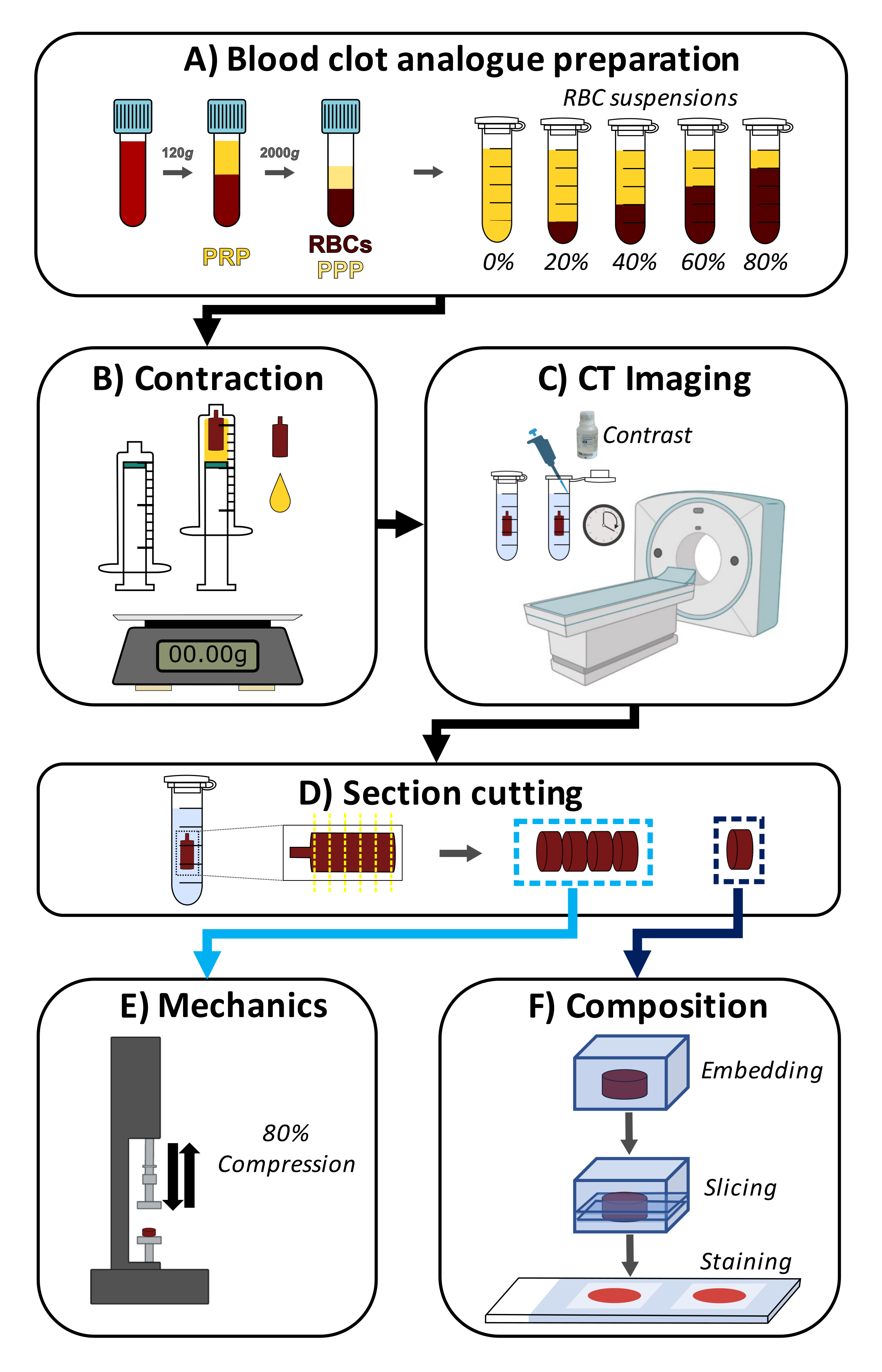

Supplement: S1 Fig — (A) Blood clot analogue preparation. Whole blood (WB) is spun in a centrifuge to separate platelet rich plasma (PRP), platelet poor plasma (PPP) and the red blood cells (RBCs). Five RBC suspensions are created using PRP with a RBC concentration of 0%, 20%, 40%, 60%, and 80%. (B) Clot contraction measurements. The empty syringe, syringe with contracted clot and serum, and separate clot or serum are weighed. (C) Computed tomography (CT) imaging. A scan of the clot in an Eppendorf tube is made, after which contrast is administered and more scans are made after specific time points. (D) The clot is cut into 5 sections: 4 for mechanical testing and 1 for composition analysis. The ends of the clot are discarded. (E) Mechanical testing. The clot is compressed up to 80% strain in a custom-made compression tester. (F) Composition analysis. The clot section is embedded and sliced in two slices. Finally, the slices are stained using H&E and the RBC content is quantified. Sections of this figure were created with BioRender.com. (TIF) [file pone.0293456.s005.tif]

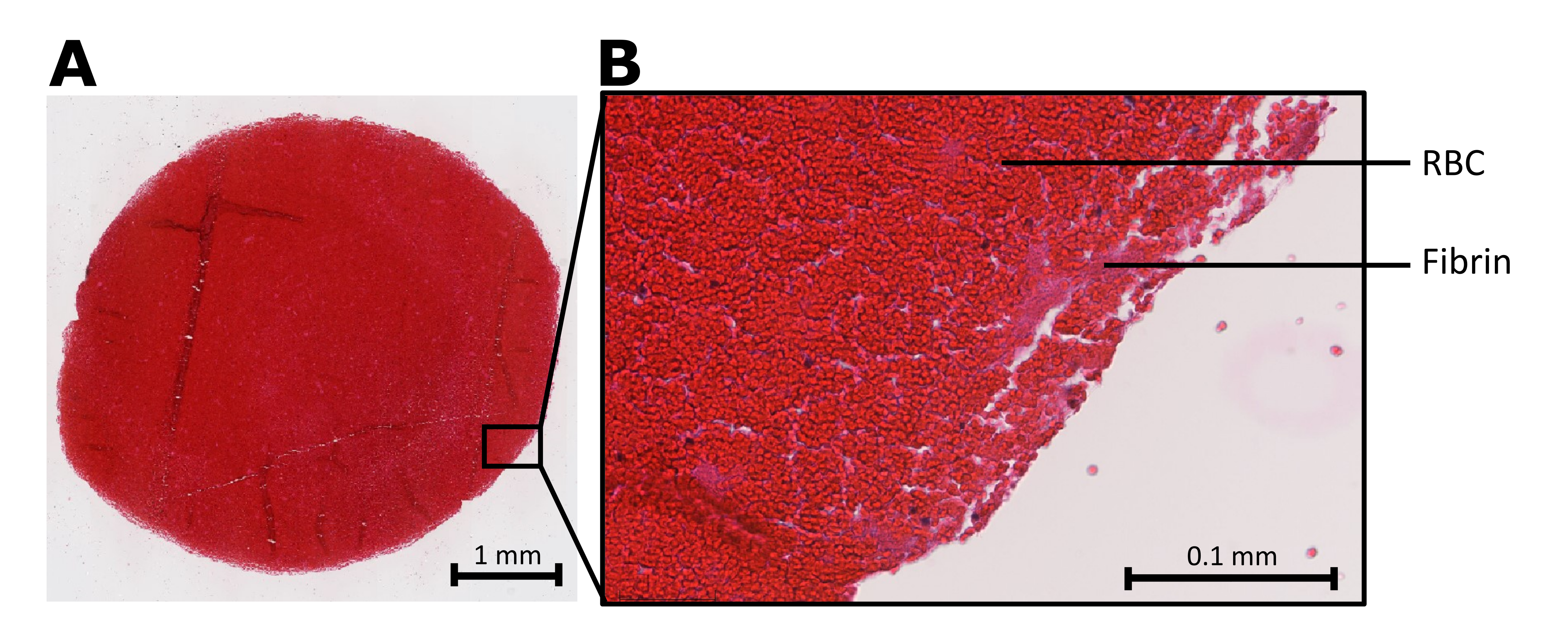

Supplement: S2 Fig — (A) An overview. (B) Magnified image which demonstrates that the clot consists primarily of RBCs; however, more fibrin tends to be present on the edge of clots compared to the internal portion. (TIF) [file pone.0293456.s006.tif]

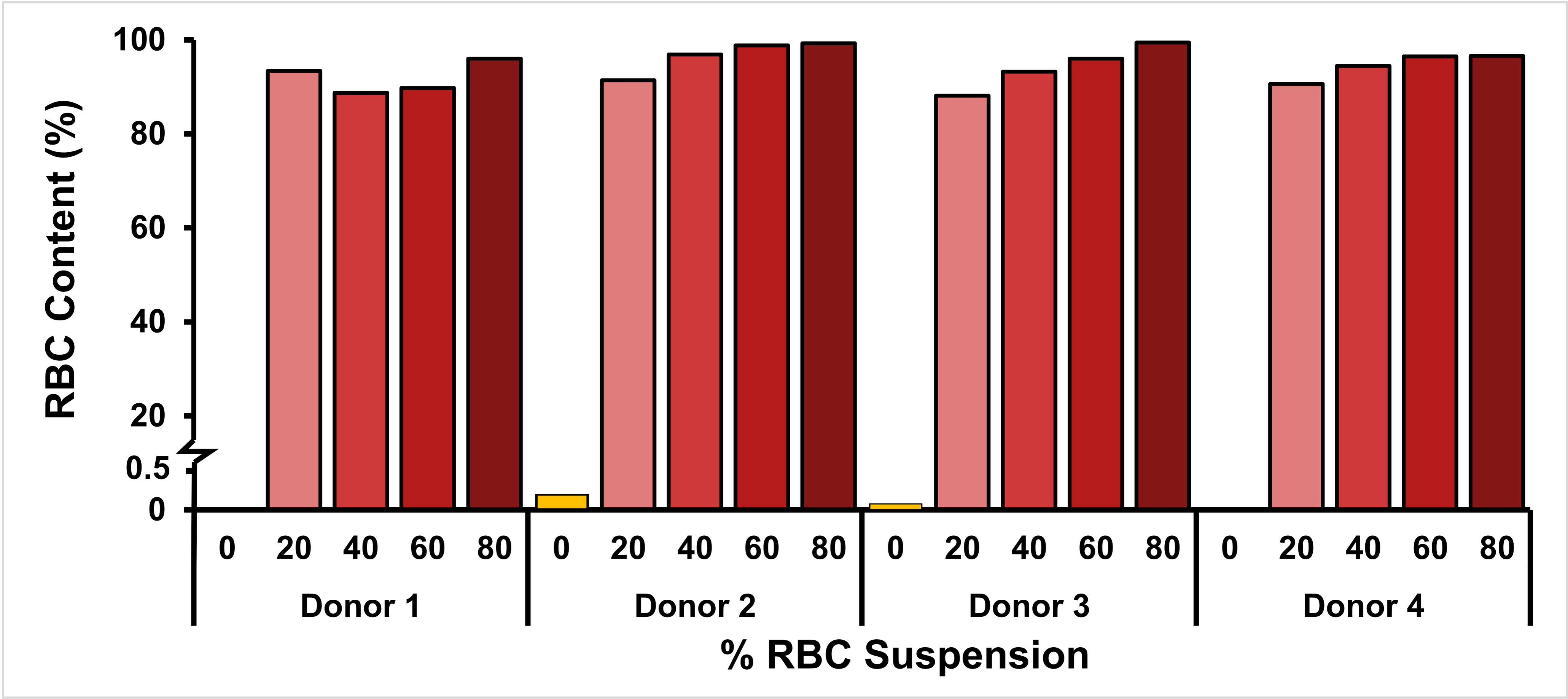

Supplement: S3 Fig — (TIF) [file pone.0293456.s007.tif]

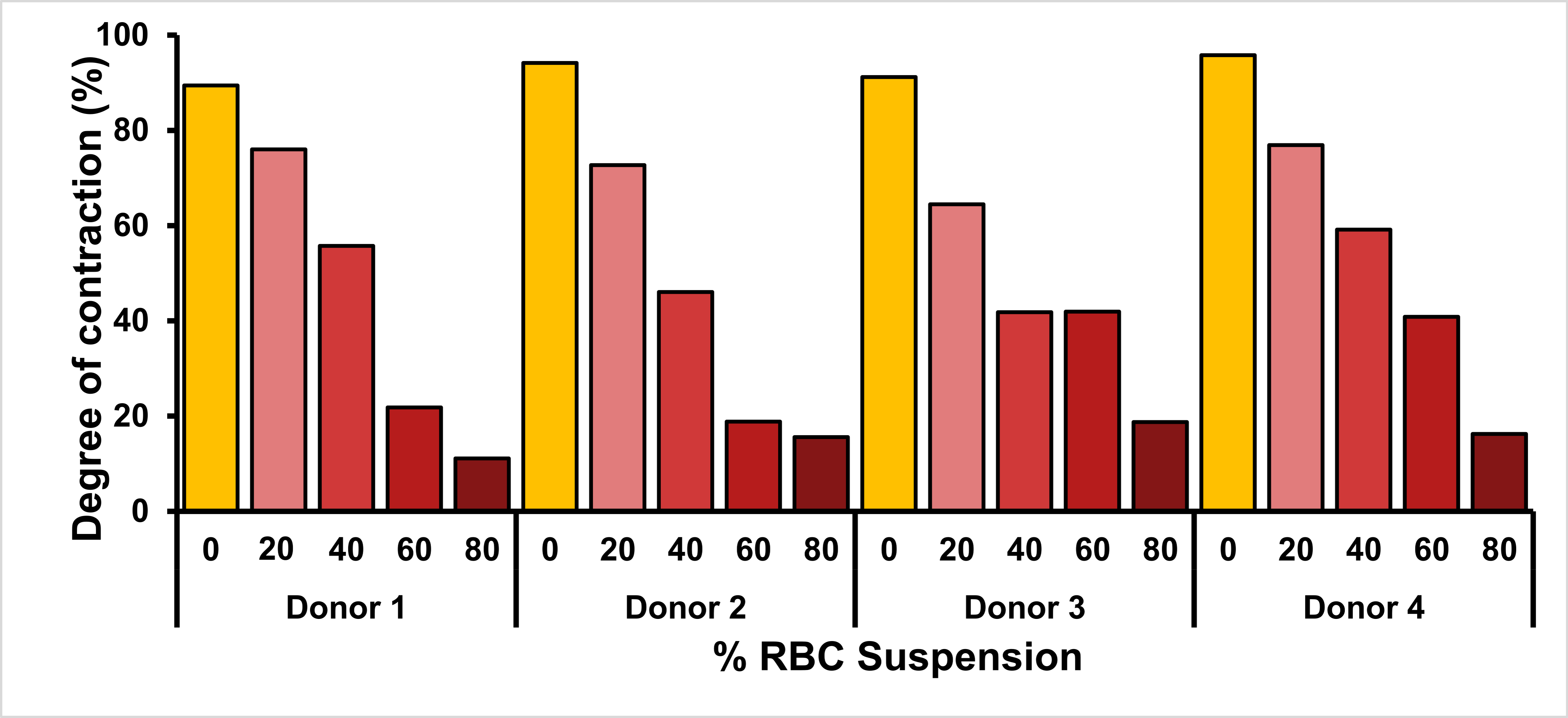

Supplement: S4 Fig — (TIF) [file pone.0293456.s008.tif]

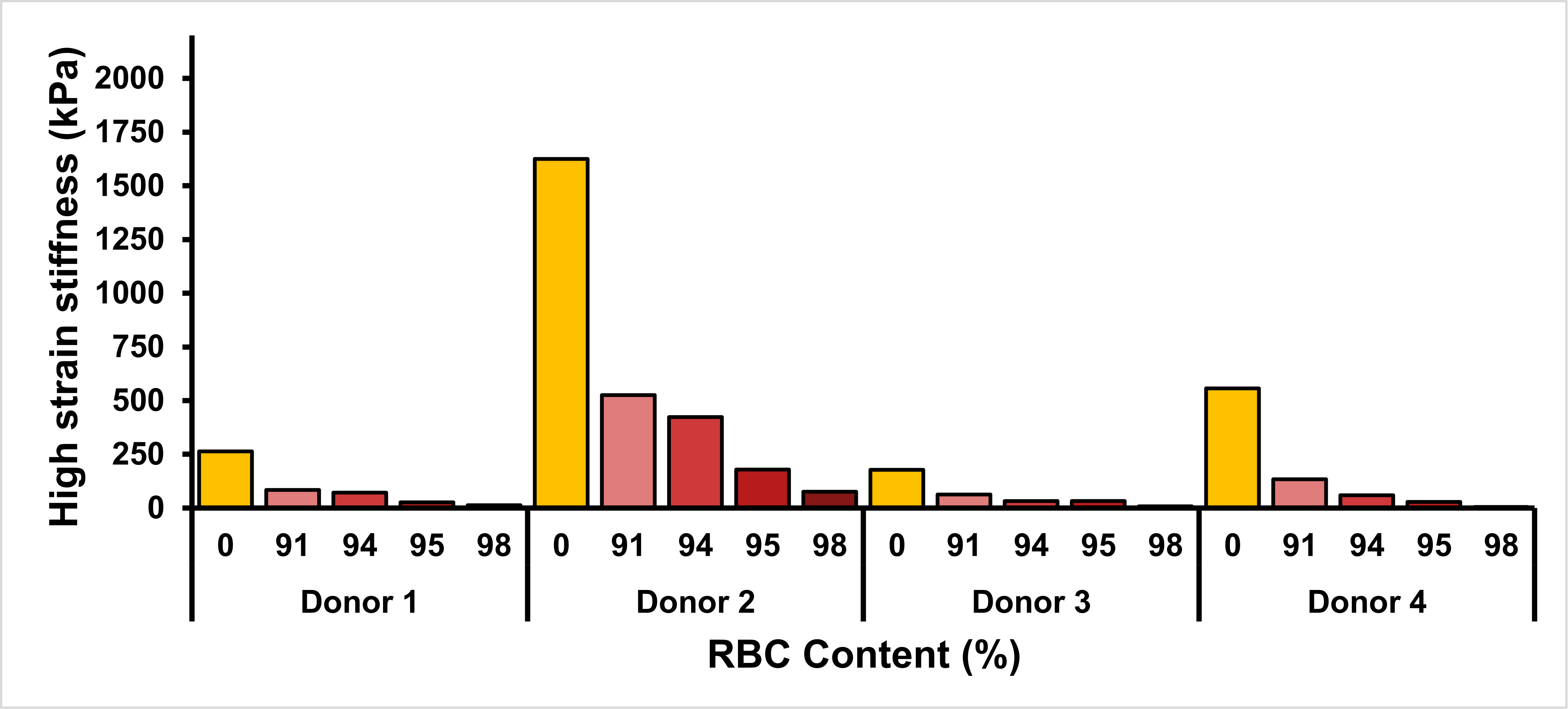

Supplement: S5 Fig — (TIF) [file pone.0293456.s009.tif]

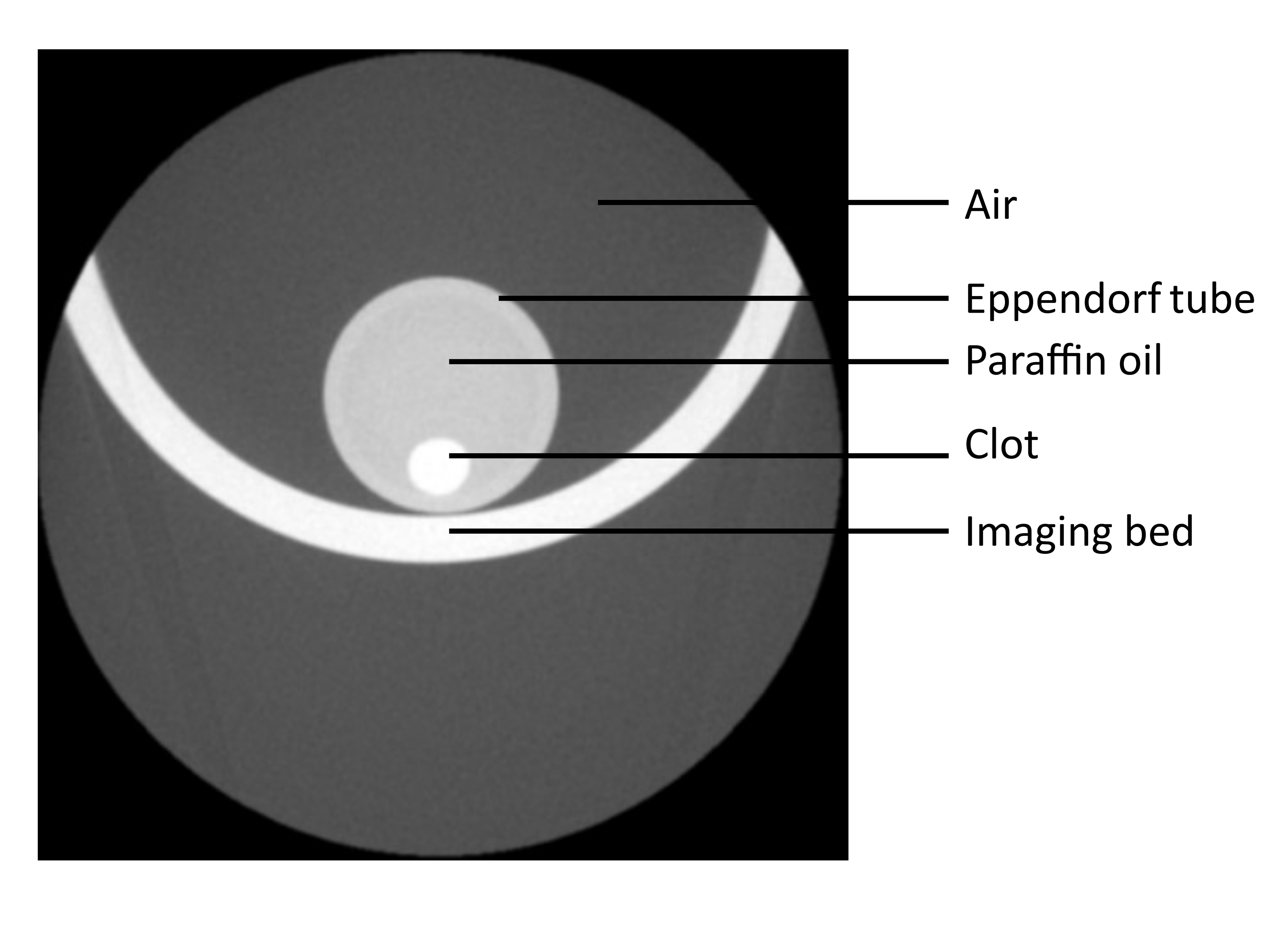

Supplement: S6 Fig — (TIF) [file pone.0293456.s010.tif]

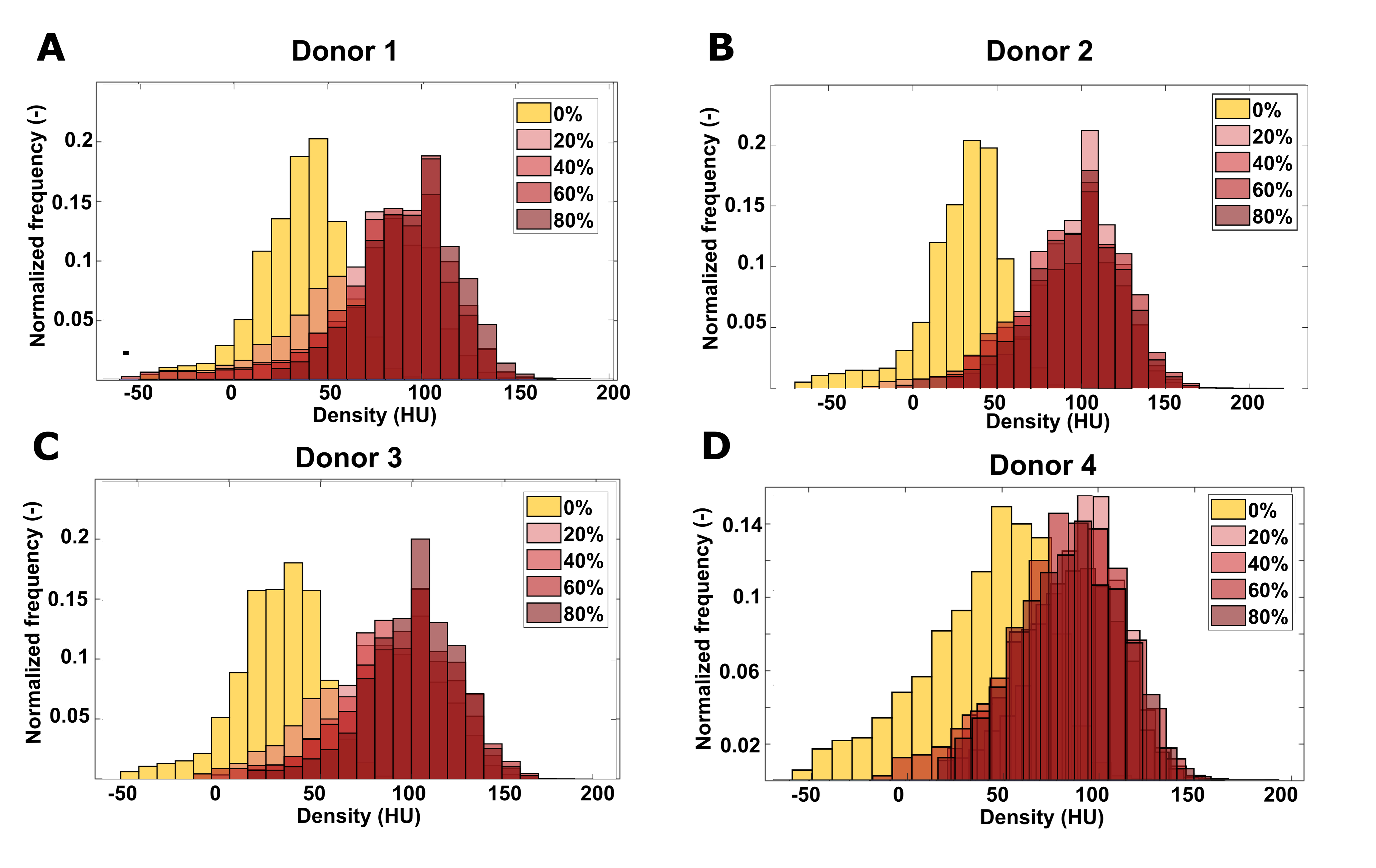

Supplement: S7 Fig — (A) donor 1, (B) donor 2, (C) donor 3, (D) donor 4. The normalized density histograms were acquired using an automatic binning algorithm based on Sturge’s rule: k = ⌈1 + log2 n⌉, where k is the number of bins and n is the number of observations. (TIF) [file pone.0293456.s011.tif]

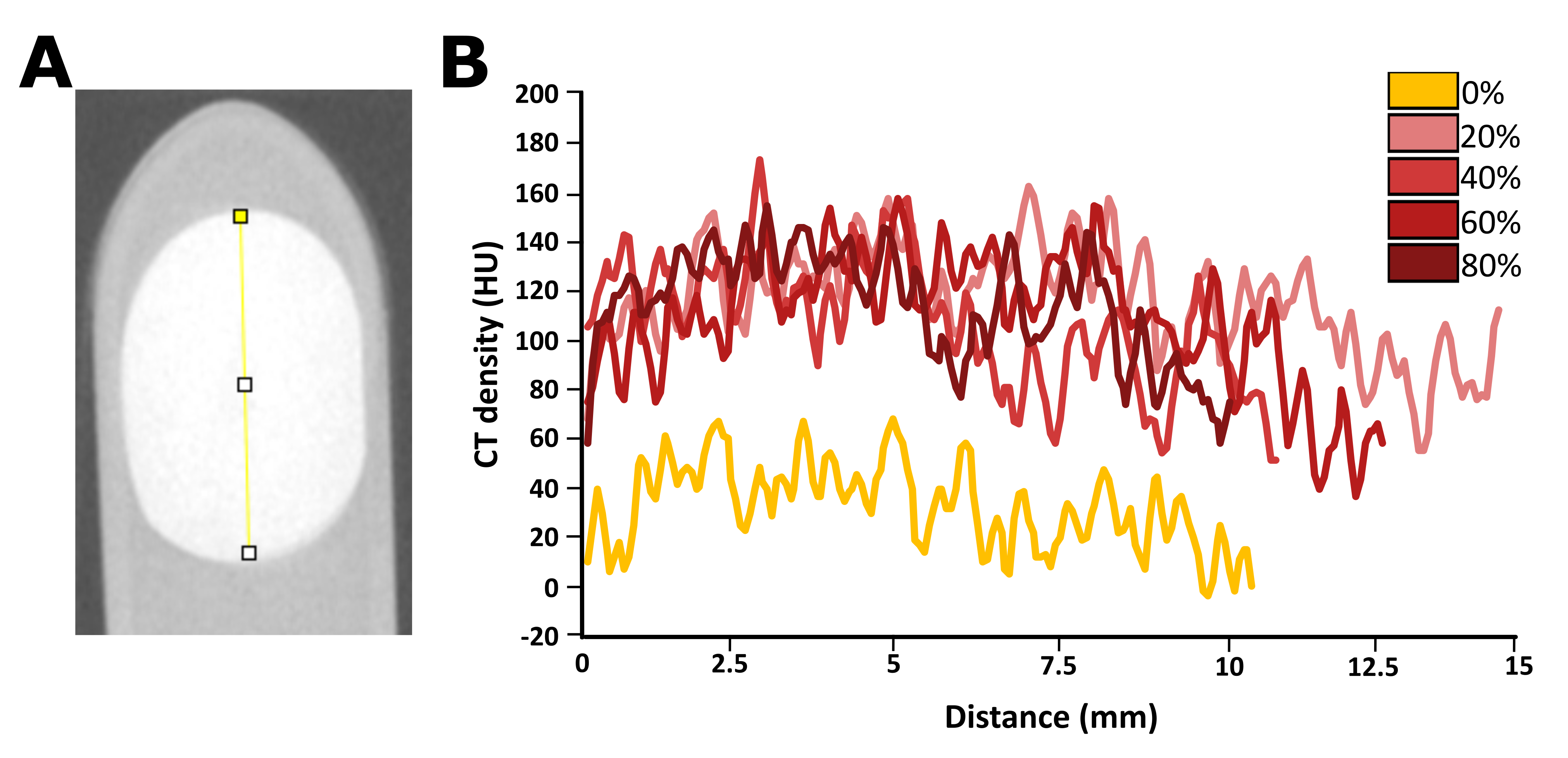

Supplement: S8 Fig — (A) Example micro-CT image of a clot with the indicated line over which the CT density is measured. (B) The distance of a clot (from top to bottom) vs the CT density for the five clot types of donor 2. (TIF) [file pone.0293456.s012.tif]

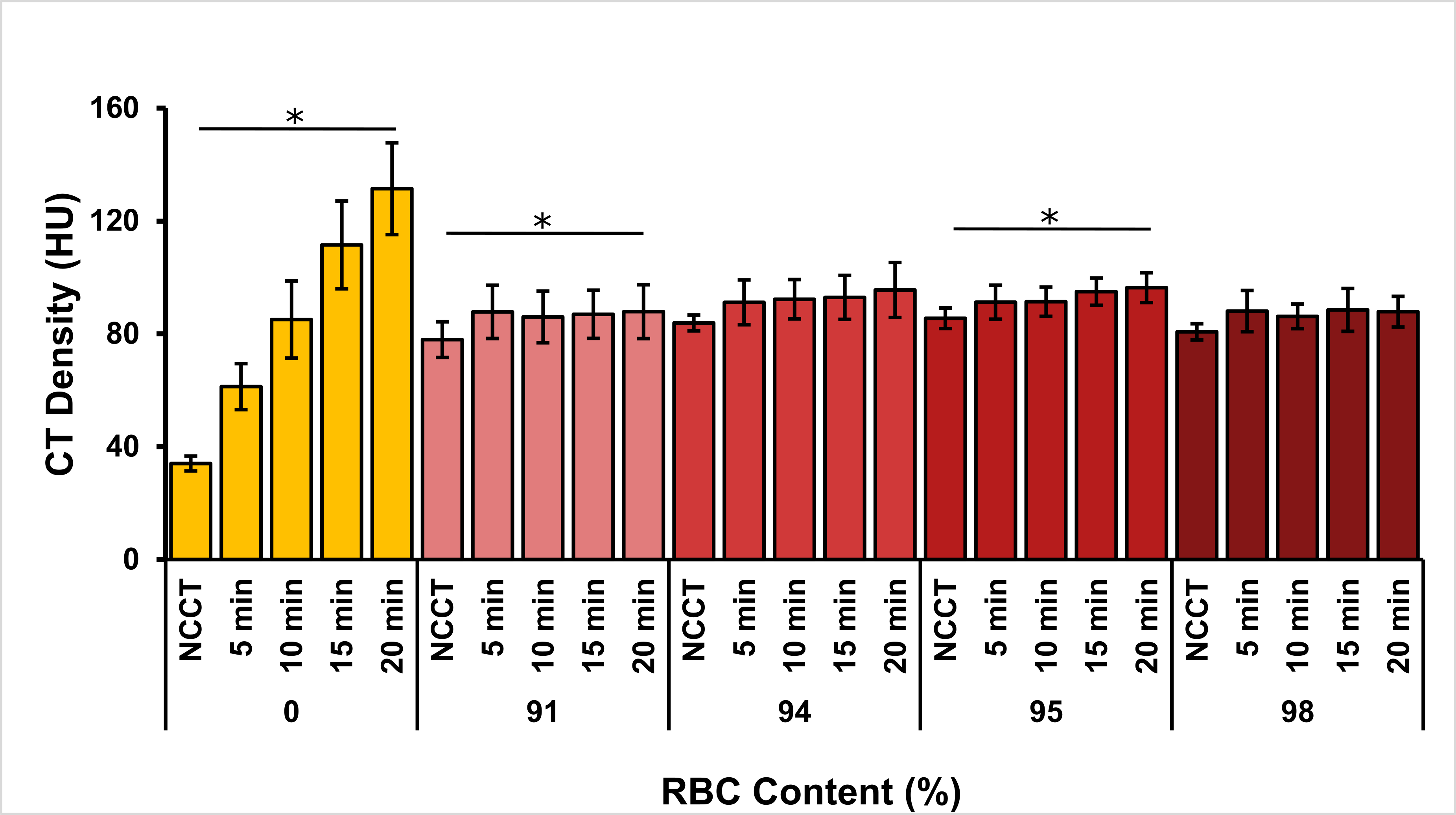

Supplement: S9 Fig — The error bars represent the standard deviation. The density significantly increased after 20 minutes of contrast exposure compared to the NCCT scan for the clots with RBC-content of 0%, 91% and 95% (P-values ranging from P = 0.003 to P = 0.043), except for the clots with RBC-content of 94% and 98% (P-value of 0.068 and 0.098, respectively). (TIF) [file pone.0293456.s013.tif]

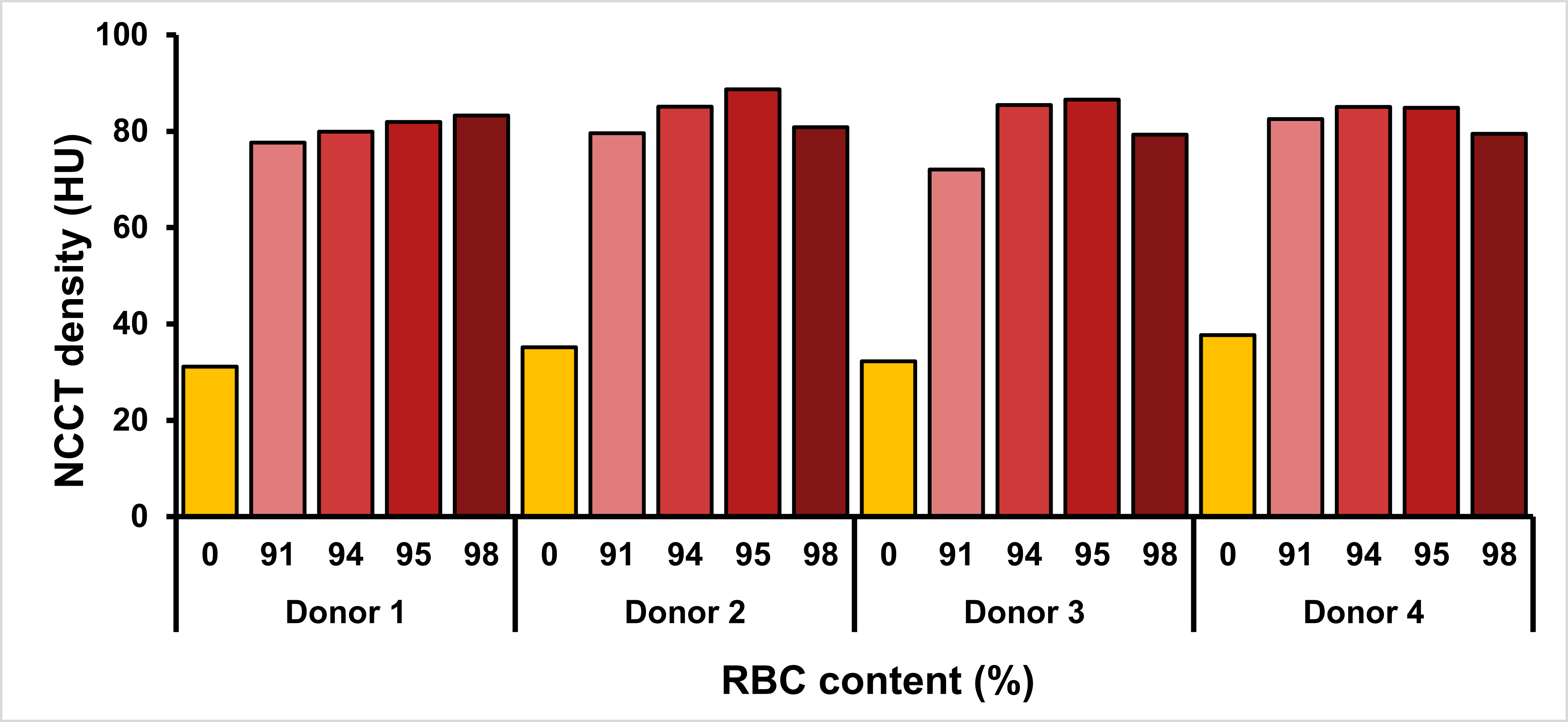

Supplement: S10 Fig — (TIF) [file pone.0293456.s014.tif]

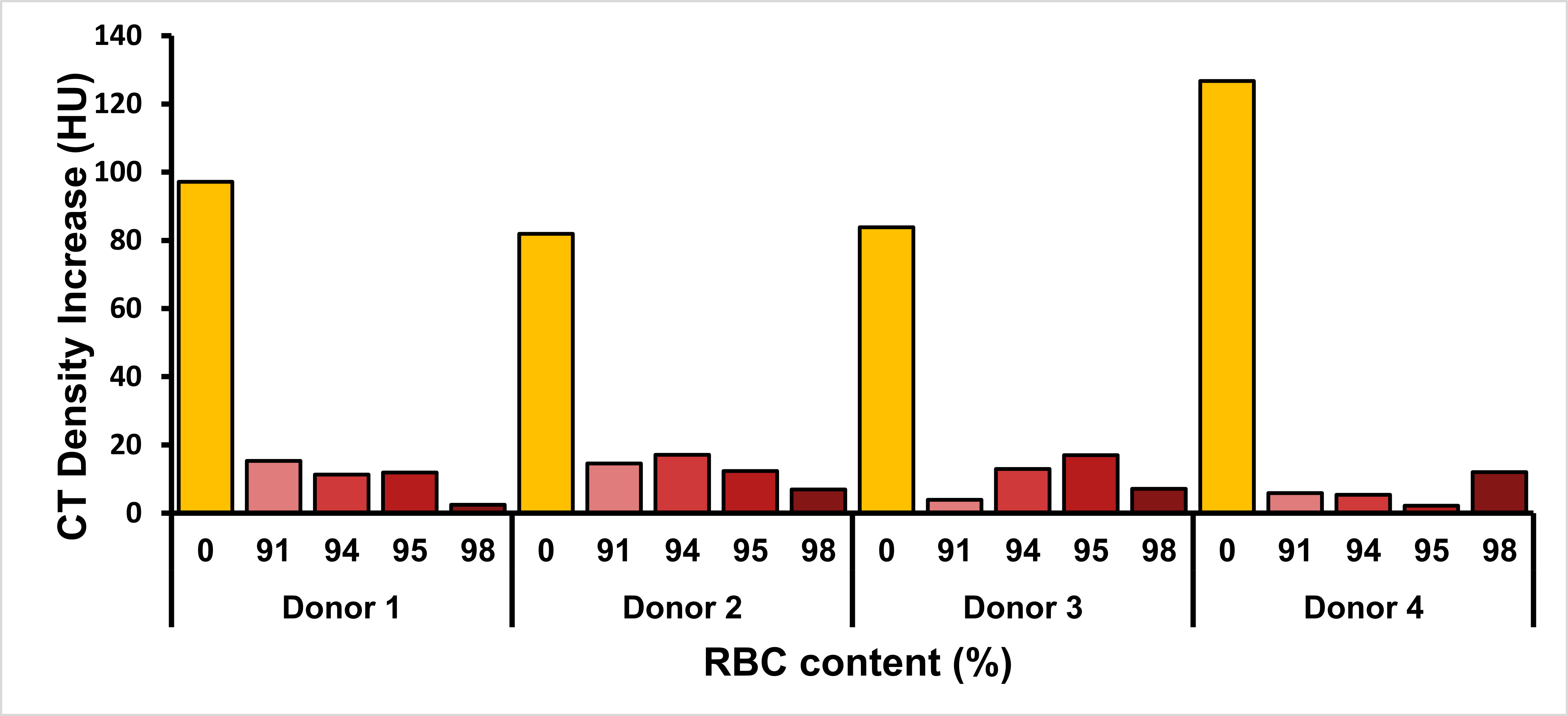

Supplement: S11 Fig — (TIF) [file pone.0293456.s015.tif]
